# Evidence for Time-of-Day Dependent Effect of Neurotoxic Dorsomedial Hypothalamic Lesions on Food Anticipatory Circadian Rhythms in Rats

**DOI:** 10.1371/journal.pone.0024187

**Published:** 2011-09-02

**Authors:** Glenn J. Landry, Brianne A. Kent, Danica F. Patton, Mark Jaholkowski, Elliott G. Marchant, Ralph E. Mistlberger

**Affiliations:** Department of Psychology, Simon Fraser University, Burnaby, British Columbia, Canada; Nagoya University, Japan

## Abstract

The dorsomedial hypothalamus (DMH) is a site of circadian clock gene and immediate early gene expression inducible by daytime restricted feeding schedules that entrain food anticipatory circadian rhythms in rats and mice. The role of the DMH in the expression of anticipatory rhythms has been evaluated using different lesion methods. Partial lesions created with the neurotoxin ibotenic acid (IBO) have been reported to attenuate food anticipatory rhythms, while complete lesions made with radiofrequency current leave anticipatory rhythms largely intact. We tested a hypothesis that the DMH and fibers of passage spared by IBO lesions play a time-of-day dependent role in the expression of food anticipatory rhythms. Rats received intra-DMH microinjections of IBO and activity and body temperature (*T*_b_) rhythms were recorded by telemetry during ad-lib food access, total food deprivation and scheduled feeding, with food provided for 4-h/day for 20 days in the middle of the light period and then for 20 days late in the dark period. During ad-lib food access, rats with DMH lesions exhibited a lower amplitude and mean level of light-dark entrained activity and *T*_b_ rhythms. During the daytime feeding schedule, all rats exhibited food anticipatory activity and *T*_b_ rhythms that persisted during 2 days without food in constant dark. In some rats with partial or total DMH ablation, the magnitude of the anticipatory rhythm was weak relative to most intact rats. When mealtime was shifted to the late night, the magnitude of the food anticipatory activity rhythms in these cases was restored to levels characteristic of intact rats. These results confirm that rats can anticipate scheduled daytime or nighttime meals without the DMH. Improved anticipation at night suggests a modulatory role for the DMH in the expression of food anticipatory activity rhythms during the daily light period, when nocturnal rodents normally sleep.

## Introduction

When food is freely available, daily rhythms of foraging, food intake and physiology are synchronized to the solar day by entrainment of a retinorecipient master circadian pacemaker, the hypothalamic suprachiasmatic nucleus (SCN) [1]. The SCN of nocturnal rodents confers circadian organization by actively promoting arousal at night and rest during the day [2], [3], and by coordinating the phase of circadian clocks in peripheral organs via neural, hormonal and behavioral signals [4]. Under standard laboratory conditions, the timing of these rhythms relative to the daily light-dark (LD) cycle is typically stable, but can be markedly altered if food is restricted to the ‘wrong’ time of day. Nocturnal rats and mice fed only in the middle of the light period, when sleep normally predominates, exhibit an inversion of the phase of circadian clocks in most peripheral organs, and the emergence of a food-anticipatory activity rhythm, evident in general activity, wheel running or operant behaviors such as lever pressing and food-bin approaches [5]–[7]. During such daytime restricted feeding schedules, the SCN pacemaker does not invert its phase [8]–[10]. Moreover, while ablation of the SCN eliminates daily rhythms in rats with free access to food, it does not affect food anticipatory behavioral and physiological rhythms, which emerge if food is temporally restricted, and then persist during total food deprivation tests lasting several days [11]–[13]. These and other properties indicate that food anticipatory behavioral rhythms are controlled by a circadian mechanism outside of the SCN that can override signals from the SCN that normally suppress daytime activity and arousal. The mechanism has been conceptualized as a food-entrainable oscillator or pacemaker, analogous to the light-entrainable SCN pacemaker [5]–[7].

Two questions that arise in neurobiological analysis of food anticipatory rhythms are where in the brain (or body) are the driving food-entrainable circadian oscillators located, and how do these overcome sleep promoting signals from the SCN to induce arousal in the usual sleep phase? Numerous brain regions exhibit daily rhythms of immediate early gene or clock gene expression that are shifted or induced by daily feeding schedules [14]. One structure in which this occurs is the dorsomedial hypothalamus (DMH) [15]–[22]. Partial lesions of the DMH created by local infusion of the excitatory neurotoxin ibotenic acid were reported to markedly attenuate food anticipatory rhythms of activity, sleep wake and body temperature (*T*_b_) rhythms in rats, supporting a conclusion that the DMH is critical for the expression of these rhythms [16]. However, efforts to support this conclusion using electrolytic and radiofrequency lesion techniques have met with failure, as rats and mice sustaining substantial or complete ablation of DMH cells and fibers of passage through this area were found to exhibit near normal food anticipatory rhythms of activity, body temperature and clock gene rhythms in other brain regions [22]–[24]. Subsequent studies ruled out several procedural variables as possible explanations for the differing results, including cage configuration, method of feeding and measure of activity [24], [25].

Circumstantial evidence suggests that the lesion method might be important. SCN outputs to sleep-wake regulatory circuits project in part either directly or indirectly to and through the DMH area [2], [26]–[28]. The SCN receives or is surrounded by fibers from hypothalamic structures that process feeding and metabolism related signals, including the DMH [29]–[30] and arcuate nucleus [31]. Expression of the immediate early gene *cFos* (a marker of neural activity) or the clock gene *per1* in the SCN can be inhibited by behavioral arousal and some metabolic signals [31]–[33]. These observations have been connected in the following way [22], [30], [34]. During restricted daytime feeding schedules, SCN sleep promoting outputs, conveyed in part by direct or indirect projections through the DMH area, might be directly inhibited by inputs from nutrient sensitive structures such as the DMH that are induced to oscillate in phase with a scheduled mealtime. DMH lesions made by neurotoxins such as ibotenic acid [16] would eliminate neurons responsible for inhibiting SCN output, and would spare SCN fibers of passage through the DMH area. These SCN outputs would then suppress food anticipatory activity without opposition. By contrast, DMH lesions made by radiofrequency current [22]–[24] would destroy not only DMH neurons inhibitory to the SCN, but also SCN outputs that project through and around the DMH area. This would allow food anticipatory activity to be expressed without opposition from the SCN, assuming that food-entrainable oscillators that drive these rhythms are located in whole or in part outside of the DMH.

This model leads to two predictions. If SCN outputs are responsible for attenuation of food-anticipatory rhythms in rats with ibotenic acid-induced DMH lesions, then food-anticipatory rhythms in these rats should be restored by 1. SCN ablation, or 2. scheduling mealtime at night, when the SCN does not inhibit activity. A recent study has confirmed the first prediction; rats with attenuated daytime food anticipatory rhythms following DMH ablation exhibited robust food anticipatory rhythms when the SCN were subsequently ablated [30]. In the present study, we followed closely the recording and ablation methods of Gooley et al [16] and provide evidence consistent with the second prediction; anticipation of a daytime meal was weak in some rats with ibotenic acid-induced DMH lesions, and was restored to levels characteristic of DMH-intact rats when the scheduled mealtime was shifted to late in the night.

## Materials and Methods

### Animals and recording apparatus

All animal work was conducted according to guidelines established by the Canadian Council on Animal Care and was approved by the University Animal Care Committee at Simon Fraser University (permit number 732P95). Young adult male Sprague Dawley rats (N = 29, Charles River, Montreal, Canada) were housed in group cages under a 12h∶12h light-dark (LD) cycle in a climate controlled vivarium (22±1°C). The rats then underwent surgical procedures to create a neurotoxic or sham DMH lesion and implant a calibrated radiotelemetry transponder (ER-4000, Minimitter Inc., OR, USA). After recovery, the rats were housed individually in standard plastic cages (45×24×20 cm) equipped with a food hopper and water bottle holder, as in Gooley et al [16] and Landry et al [24]. Each cage was placed on top of an ER-4000 radiotelemetry receiver, housed inside individual sound-attenuating recording chambers with controlled lighting and an exhaust fan (Lafayette Instruments, IN, USA). Radiotelemetry signals were converted by the VitalView data acquisition system (Minimitter, Inc.) into pulses at a rate proportional to *T*_b_. Changes in signal strength caused by movement of the rat were converted to pulses proportional to locomotor activity. Pulses were summed and stored in 1 minute time bins.

### Surgical procedures

Rats were anesthetized for stereotaxic surgery using ketamine (90 mg/kg), xylazine (9 mg/kg), and isoflurane (0.5% to 2.0%). Following Gooley et al [16], ibotenic acid (Sigma) was dissolved in phosphate buffered saline (PBS) at a concentration of 10%, using 6N NaOH, with pH adjusted to ∼7.2 using 6N HCl. In pilot tests, concentrations of 1–2% produced very little DMH damage, while infusions into the hippocampus caused extensive cell loss, demonstrating bioactivity of the neurotoxin at these concentrations. At the 10% concentration, bilateral infusions into the DMH were associated with immediate and severe respiratory depression. A two-stage lesion procedure was therefor adopted, with ∼4 weeks between unilateral 300 nL (N = 8 rats) or 100 nL (N = 6 rats) infusions delivered by Hamilton syringe, using the following stereotaxic coordinates relative to bregma (10% angle): 3.4 mm posterior, ±2.2 mm lateral from midline, −7.7 mm ventral from dura. Seven additional rats received vehicle injections, and 8 rats served as unoperated controls. Radiotelemetry transponders were implanted ∼7 days after the second stereotaxic surgery.

### Feeding schedules

Continuous recording of activity and *T*_b_ in the temporal isolation chambers began 2 weeks after the transponder implants. The rats received food (rodent chow pellets, 5001) *ad-libitum* in LD 12∶12 for 15 days, with one day in constant dark (DD; day 10). Food was removed for 42 h, and then provided for 4 h daily, beginning 6 h after lights-on (zeitgeber time 6, where ZT0 is defined as lights-on, by convention). After 20 days, the lights were turned off for 3 days of DD. Food was provided at the usual time on the first DD day, and was removed for the final 50 h. Food was then provided *ad-libitum* for 19 days, removed for 38 h and then provided for 4 h each night, beginning 9 h after lights-off (ZT21). The lights were turned off for 3 days, a last meal was provided at ZT21, and the rats were then food deprived for the last 60 h of constant dark. Food was then available *ad-libitum* for 5 days, removed for 54 h, and then provided for 4 h each day at ZT6, for 9 days. The rats then received an overdose of pentobarbitol (Euthanol) and were processed for histological analysis of the lesions.

### Histological analysis

The rats were perfused transcardially with 50 ml of 0.1 M phosphate buffered saline (PBS, pH∼7.3) followed by 50 ml of 4% paraformaldehyde in PBS (PFA, pH∼7.3). Brains were removed, postfixed in PFA, cryoprotected in 20% sucrose overnight, and then frozen sectioned at 40 µm in a cryostat for histological confirmation of the lesion site. Sections were mounted on slides and stained for Nissl with cresyl violet. Brain sections were evaluated using a Nikon Eclipse 80i light microscope. The extent of the lesions was assessed based on loss of neurons and presence of gliosis. Templates of the DMH were created based on the Paxinos and Watson rat brain atlas and published photomicrographs [29], [35]. The DMH area was divided into quadrants, with each box representing 12.5% of total DMH area per section. The amount of undamaged DMH tissue in each box was estimated as a percentage of volume.

### Activity and *T_b_* analyses

Activity and *T*_b_ data were evaluated in the form of raster plots displaying all days and waveforms averaged over blocks of days using Clocklab (Actimetrics, Inc., IL), Circadia (Dr. T. A. Houpt, Florida State U, USA), and Prism (Graphpad Software, Inc., San Diego) software. Food anticipation was quantified by expressing activity during the 3 h prior to mealtime (ZT3-6) as a ratio relative to total daily activity [16] or to activity from ZT12-ZT3 [24]. The two methods produced equivalent results, therefore only the latter is presented here. The amplitude of the day-night rhythm of activity was expressed as a nocturnality ratio by dividing 12 h nightime activity by the 24 h daily total. The amplitude of the *_Tb_* rhythm was expressed as a difference between the mean at night and the mean in the day. Group means of activity and *_Tb_* data were calculated separately for the high IBO dose rats, the low IBO dose rats, and the DMH intact control groups (unlesioned and sham operated rats combined). Group differences were evaluated statistically using 2-way repeated measures ANOVA (Prism 5.0, Graphpad Software) and planned t-tests.

## Results

### DMH lesions

**Figure 1:**
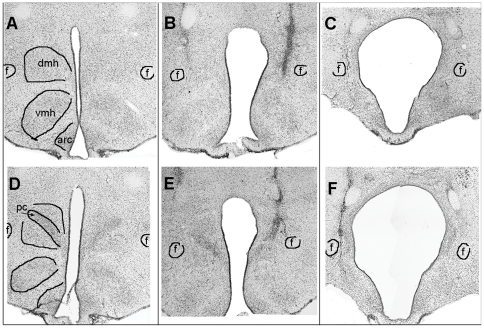
Digital images of 40 µm cresyl violet stained coronal brain sections through the hypothalamus at two levels of the dorsomedial hypothalamic nucleus (DMH). A–C: ∼2.9 mm posterior to bregma (Paxinos and Watson, 2009). D–F: ∼3.24 mm posterior to bregma. (A,D). An intact rat. (B,E). A rat sustaining minor (<20%) DMH damage. (E,F). The rat sustaining the largest lesion, with complete ablation of the DMH, including the DMH pars compacta (PC), and partial damage to the ventromedial hypothalamic nucleus (VMH) and the arcuate nucleus (ARC).

The hypothalamus of rats that received IBO infusions presented with an expanded 3^rd^ ventricle and loss of identifiable DMH tissue ranging from minor (estimated to be = <15%, e.g., Fig. 1B) to severe (estimated to be 100%; e.g., Fig. 1C). In the most severe case (Fig. 1C), the 3^rd^ ventricle extended laterally to within ∼200 µm of the fornix, dorsally to the mammilothalamic tract, and ventrally to the arcuate nucleus. The ventromedial hypothalamus was absent on one side and reduced by 50% contralaterally. The medial 50% of the arcuate was also destroyed. Tissue surrounding the border of the lesion was heavily gliosed. The large size of the lesion cavity likely reflects the extended post-lesion survival time, sufficient to permit removal of cellular debris. Fibers of passage spared by the neurotoxin presumably run through intact and gliosed tissue surrounding the expanded 3^rd^ ventricle. Most of the other rats receiving the 300 nl volume injections also exhibited extensive damage to the DMH, but in each case some of the DMH pars compacta could be identified at least unilaterally in at least 2 sections, and total surviving DMH volume was estimated to range from 20% to 80% across rats. Rats receiving the 100 nl volume injections showed much smaller lesions, characterized by some expansion of the 3^rd^ ventricle, and damage estimated to be 20% or less.

### Activity during ad-lib food access and food deprivation

**Figure 2:**
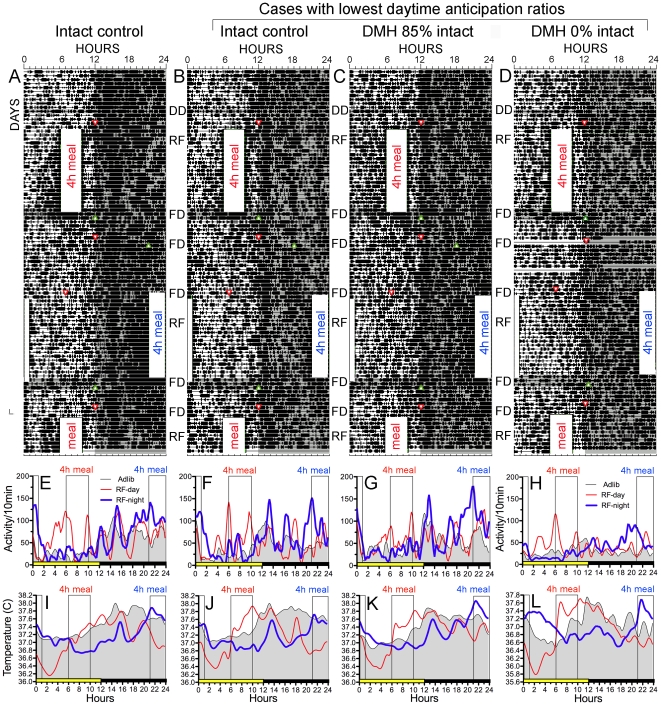
Activity and body temperature rhythms in representative intact and DMH-lesion rats. Raster plots of locomotor activity (A–D), and average waveforms of locomotor activity (E–H) and body temperature (I–L) from an intact control rat with strong daytime food anticipatory rhythms (A,E,I), an intact rat with the weakest daytime food anticipation (B,F,J; defined by the lowest food anticipation ratio during daytime feeding), the rat with the lowest daytime food anticipation ratio (C,G,K) from the group that received a low-dose ibotenic acid microinjection causing a small partial lesion, and the rat with the lowest daytime food anticipation ratio (C,G,K) from among rats receiving a high-dose lesion that induced a complete DMH lesion (Fig. 1C,F). The raster plots illustrate activity summed in 10 min bins. Each line represents 24 h of recording, with time of day plotted from left to right (144 time points/day), and consecutive days aligned vertically. Time bins in which activity counts were registered are represented by a heavy bar, the height of which signifies the amount of activity (1–10 counts, 11–20 counts and >20 counts/10 min). Grey shading denotes lights-off (hours 12–24 of LD, or all day during constant dark tests). Scheduled mealtime during food restriction are denoted by opaque columns labeled ‘4-h meal’, with red signifying a daytime meal (hours 6–10 of the light period) and blue signifying a late nighttime meal (hours 21–01). Small red arrowheads pointing down denote beginning of total food deprivation test, and small green arrowheads pointing up denote the end of food deprivation. Waveforms of activity (E–H) and temperature (I–L) data were creating by averaging across 5 days of ad-lib food access prior to food restriction (grey shaded waveforms), the last 5 days of the daytime feeding schedule (light weighted red lines) and the last 5 days of the nightime feeding schedule (heavy weighted blue lines). 4-h mealtimes are denoted by the translucent columns. The daily lights-on period is denoted by the yellow horizontal bar above the x-axis. To improve the clarity of the waveforms, the data were subjected to a second order smoothing polynomial averaging across 4 neighbouring data points (Prism 5.0 for Mac OS X). Raw (unsmoothed) data were used for the raster plots and statistical analyses.

**Figure 3:**
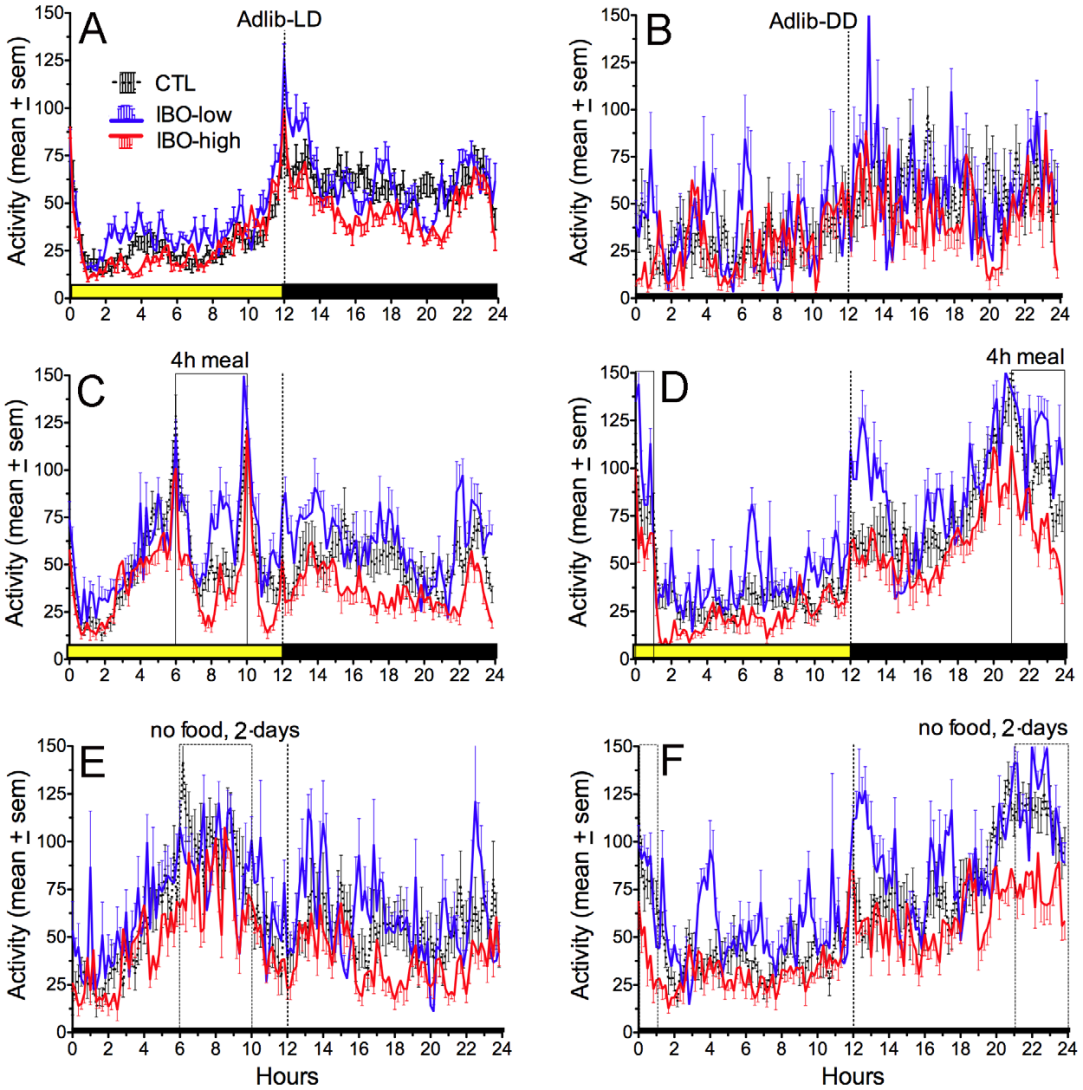
Group mean waveforms of activity data in intact controls rats (black dotted curves with standard error bars above and below the means), and in rats receiving small partial DMH lesions created by low-dose ibotenic acid (IBO) microinjections (blue curves with error bars above the means) or larger lesions created by high-dose IBO injections (red curves with error bars below the means). A. Ad-lib food access in a LD cycle, with the light period (hours 0–12) denoted by the heavy yellow bar above the x-axis. B. Ad-lib food access during a day in constant dark. C. Average of the last 5-days of daytime restricted feeding, with the daily 4-h mealtime (hours 6–10 of lights-on) denoted by the translucent column. D. Average of the last 5-day of nightime restricted feeding (hours 21–01). E. Average of days 2–3 of total food deprivation following the daytime feeding schedule. F. Average of the days 2–3 of total food deprivation following the nightime feeding schedule.

Based on group differences in lesion size, group means were calculated separately for rats receiving high-dose and low-dose IBO lesions. Raster plots and average waveforms of activity data from 4 rats are illustrated in Figure 2, to represent an intact rat with a strong food anticipatory activity rhythm (Fig. 2A), and the rat from each of the three groups that had the lowest magnitude daytime food anticipatory rhythms, based on food anticipation ratios (control rat, Fig. 2B; low-dose IBO rat with ∼85% DMH intact, Fig. 2C; high-dose IBO rat with no DMH detectable, Fig. 2D.). Group average waveforms for each feeding condition are illustrated in Figure 3.

**Figure 4:**
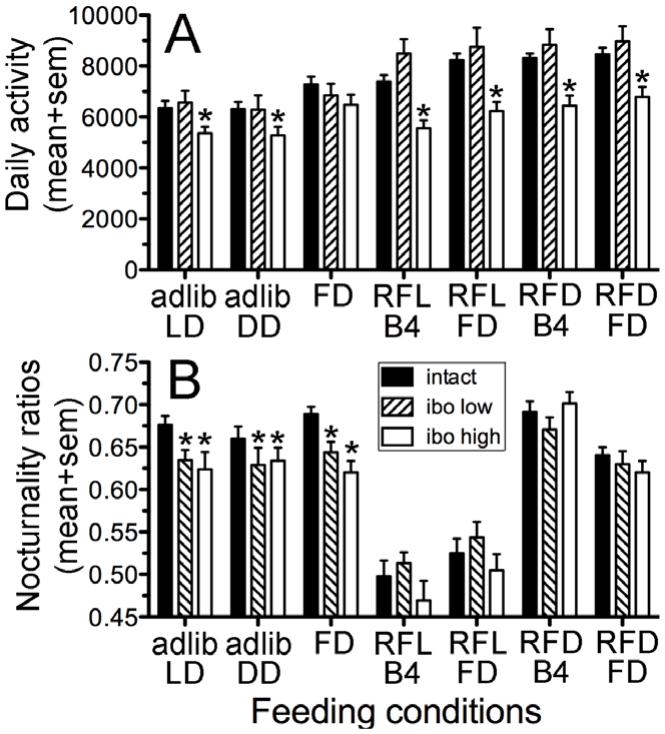
Total daily activity and nocturnal activity in intact and DMH lesion rats. A. Group mean daily activity counts in intact rats (solid black bars), low-dose ibotenic acid lesion rats (stripped bars) and high-dose lesion rats (white bars), during adlib food access in LD and constant dark (DD), during 2 days of total food deprivation (FD) prior to daytime restricted feeding, during the last 5-day block of the 20-day daytime restricted feeding (RFL-B4), during total food deprivation after daytime restricted feeding (RFL-FD), during the last 5-day block of nightime restricted feeding (RFD-B4) and during the food deprivation that immediately following nighttime restricted feeding (RFD-FD). B. Nocturnality ratios of activity data from each group under the same 7 food access conditions as in Panel A. Significant differences (p<.05) relative to the intact group within each condition are denoted by a star.

Mean daily activity levels were compared during ad-lib food access (in LD and DD), restricted feeding (the last 5-day block of daytime and nightime feeding) and total food deprivation (2-day blocks prior to restricted feeding and after both daytime and nightime feeding schedules), for a total of 7 feeding conditions. There was a significant main effect of lesion group (F_(2,27)_ = 8.96, p = .001) and feeding condition (F_(6,162)_ = 34.21, p<.0001; Fig. 4A). Relative to control rats, high-dose IBO rats exhibited significantly lower mean daily activity levels during all of the feeding conditions (p<.05 for each between-group contrast), with the exception of the first food deprivation test prior to restricted feeding. Activity levels in low-dose IBO rats were more variable and did not differ from the intact group, but trended to be higher than the high-dose IBO group. During the food deprivation tests and the last 5-day blocks of daytime and nightime restricted feeding, mean daily activity levels were increased in all groups relative to ad-lib food access (p<.05 for each within-group contrast), with the exception of the daytime restricted feeding in the high-dose IBO rats.

Nocturnality ratios for locomotor activity also differed significantly by group (F_(2,27)_ = 6.62, p<.005; Fig. 4B) and by feeding condition (F_(6,162)_ = 86.91, p<.0001), with a significant interaction (F_(12, 162)_ = 1.88, p = .04). The high-dose and low-dose IBO groups did not differ, but compared to the control rats, both groups exhibited a significantly lower nocturnality ratio (percent of total daily activity occuring during the usual dark period) during ad-lib food access in LD and DD and during the 42 h food deprivation prior to restricted feeding (p<.05). The group differences in the other conditions were not significant. Within groups, nocturnality ratios were not altered during the 42 h food deprivation. Nocturnality ratios greatly decreased during daytime restricted feeding, and during the total food deprivation test that immediately followed, consistent with the presence of a persisting rhythm of daytime food anticipatory activity in all 3 groups.

### Food anticipatory activity

All rats exhibited food anticipatory activity during both the daytime and the nighttime restricted feeding schedules, but daytime anticipation was weak in some DMH ablated rats (e.g., Fig. 2G,H), and more robust at night in all rats. Waveforms of activity averaged in 5-day blocks showed that during restricted feeding, all rats exhibited a progressive increase of activity during the 3 h prior to mealtime, with maximal values reached during the 30 min immediately preceeding mealtime (Figs. 2,3). Turning the lights off during the last day of scheduled feeding had no effect on the timing or amount of anticipatory activity.

Food anticipation was quantified by calculating ratios of activity during the 3 h prior to mealtime relative to activity during the rest of the day (excluding mealtime to lights-off, ZT6-12, when activity is partly evoked by delivery and removal of food, rather than spontaneous). Ratios were averaged in 5-day blocks during *ad-libitum* food access and the two 20-day feeding schedules in LD. Low- and high-dose IBO groups exhibited similar average waveforms and ratios and were therefor combined to increase power for statistical contrasts with the intact rats. Repeated measures ANOVA revealed a significant effect of time block (F_(11,286)_ = 127.8, p<.0001) and a significant interaction between time block and group (F_(22,286)_ = 2.45, p = .0004) but no effect of group alone (F_(2,26)_ = 2.69, p = .09). A series of planned one-tailed t-tests (statistically liberal) was also conducted to contrast anticipation ratios between the high-dose IBO and control group alone, for each of the 4 blocks of daytime and nighttime feeding schedules, but no group differences were significant at p<.05, even without bonferroni correction for multiple tests.

**Figure 5:**
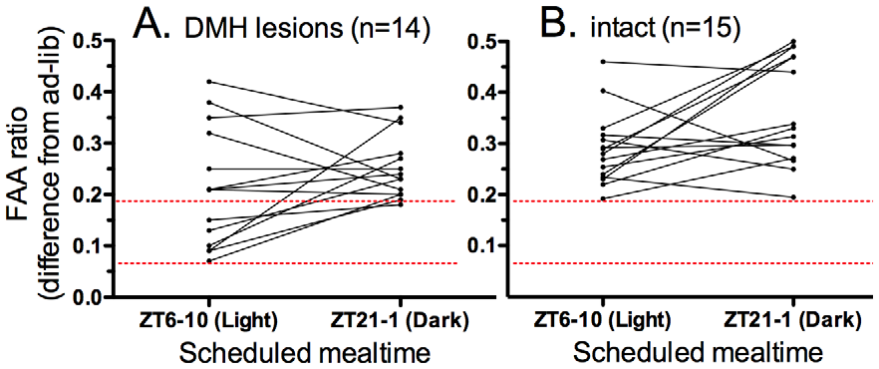
Food anticipatory activity (FAA) ratios for each DMH lesioned (A) and intact (B) rat, averaged over the last 5 days of daytime restricted feeding (ZT6-10 meal) and nightime restricted feeding (ZT21-1 mealtime). Ratios are calculated by dividing total activity during the 3-h immediately preceding mealtime by total activity during the 12-h night plus the first 3-h of the light period, and are expressed as difference scores from the same ratios calculated for the ad-lib food access condition. Data points from individual rats are conncected by lines. The upper and lower red dotted lines denotes the lowest anticipation ratios in the intact rats and the lesion rats, respectively.

Although the group mean anticipation ratios did not differ during restricted feeding, the 6 rats with the lowest magnitude ratios during the daytime feeding schedule (expressed as differences from the ratio calculated for the *ad-libitum* food access baseline block) were all among the DMH lesion groups (Fig. 5A). In each case, when mealtime was shifted to the night, the magnitude of the anticipation ratio increased to within the range exhibited by intact rats (Fig. 5B). The improvement from daytime to nighttime food anticipation is best illustrated by the rat that sustained the largest DMH lesion (100% complete) following high-dose IBO (Fig. 2D,H). This rat exhibited the weakest anticipation of the daytime meal (defined as the lowest anticipation ratio during the last 5-day block of restricted feeding), but much stronger anticipation of the nightime meal, at a level comparable to an intact rat.

The remaining 8 rats with DMH lesions showed daytime food anticipation ratios within the range exhibited by intact rats, and on average did not show enhanced anticipation ratios during nocturnal restricted feeding. All of the DMH lesions in these rats were partial, and tended to be smaller and less symmetrical than the lesions in the 6 rats with the lowest anticipation ratios. However, due to a wide range of lesion size within groups, the difference was not statistically significant (25±8% vs 41±26% complete, *t*_(12)_ = 1.08, *p* = 0.29).

Anticipation rhythms evident during the two feeding schedules persisted during 2 days of total food deprivation tests in DD (Fig. 3E,F). This was confirmed by inspection of average waveforms and by anticipation ratios for all individual rats. The magnitude of persistence appeared weaker following the nighttime restricted feeding in the high-dose IBO group, but this apparent difference was not statistically significant compared to the other groups.

Following the nighttime restricted feeding schedule and a week of ad-lib food access the rats were subjected to a 2-day food deprivation and a second and final daytime feeding schedule for 9 days. Food anticipation ratios during the last 5 days of this schedule did not differ from ratios during the second 5 day block of the first daytime feeding schedule, indicating that higher anticipation ratios during nighttime restricted feeding were not due a gradual recovery over time.

### Body temperature

**Figure 6:**
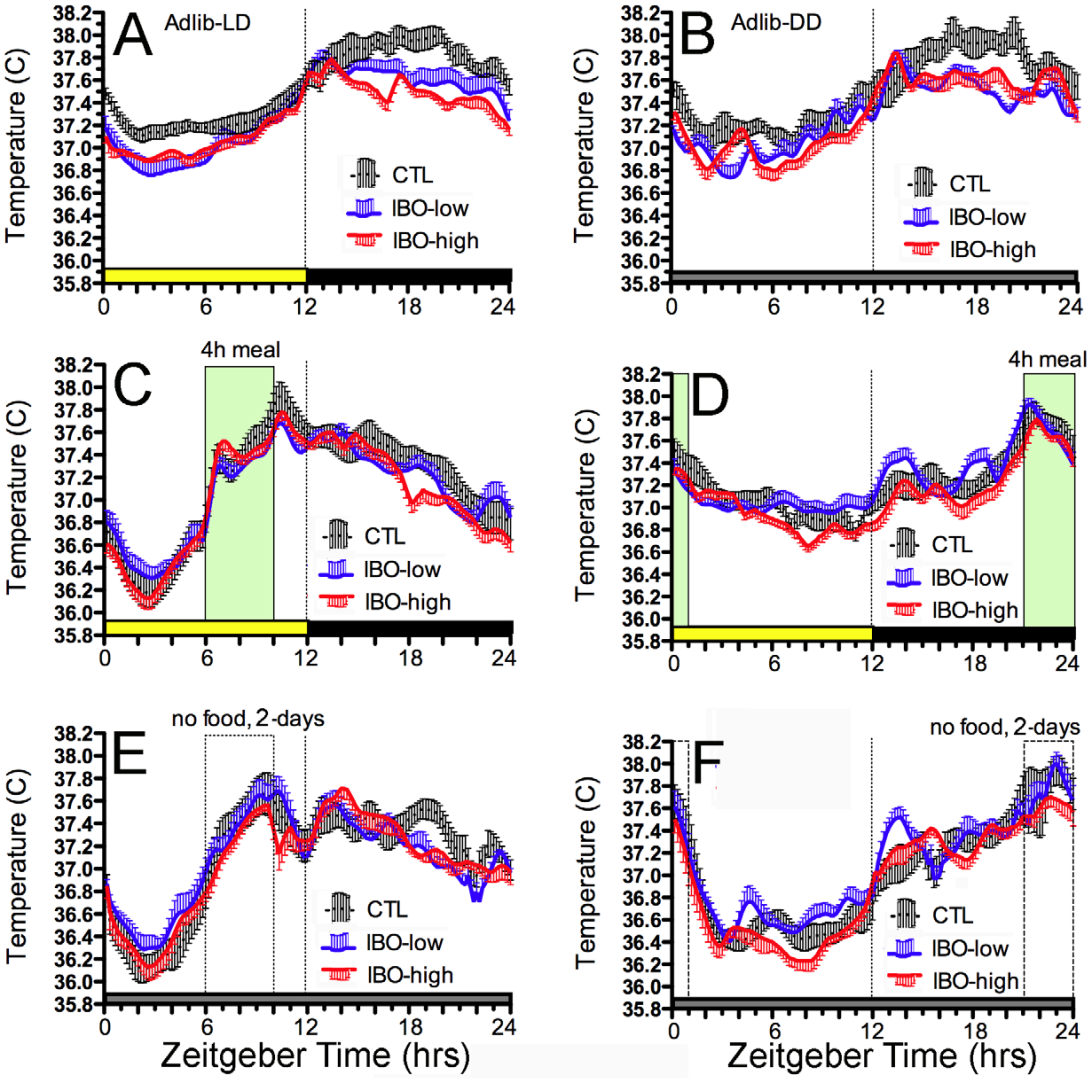
Group mean waveforms of body temperature (*T_b_*) data in intact controls rats (black dotted curves with standard error bars above and below the means), and in rats receiving small partial DMH lesions created by low-dose ibotenic acid (IBO) microinjections (blue curves with error bars above the means) or larger lesions created by high-dose IBO injections (red curves with error bars below the means). A. Ad-lib food access in a LD cycle, with the light period (hours 0–12) denoted by the heavy yellow bar above the x-axis. B. Ad-lib food access during a day in constant dark. C. Average of the last 5-days of daytime restricted feeding, with the daily 4-h mealtime (hours 6–10 of lights-on) denoted by the translucent column. D. Average of the last 5-day of nightime restricted feeding (hours 21–01). E. Average of days 2–3 of total food deprivation following the daytime feeding schedule. F. Average of the days 2–3 of total food deprivation following the nightime feeding schedule.

**Figure 7:**
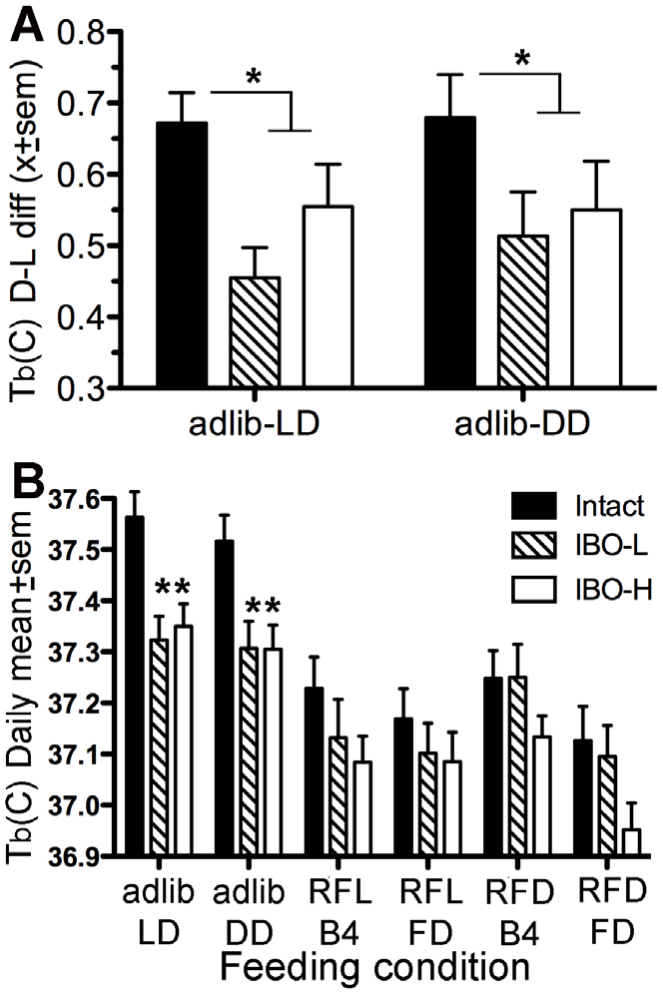
Group mean body temperatures in intact rats (black bars), low-dose DMH lesion rats (stripped bars) and high-dose lesion rats (white bars), expressed as A. dark-light differences during ad-lib food access in LD and DD, and as B. 24-h daily means during each of 7 feeding conditions. Abbreviations: RFL, last 5 days of restricted feeding in the light period; RFD, last 5 days of restricted feeding in the dark period; FD, 3-days of total food deprivation immediately following RFL or RFD.

During *ad-libitum* food access in LD, *T*_b_ was higher at night than during the day in all rats (Fig. 2I–L; Fig. 6A). The day-night *T*_b_ difference was significantly greater in the intact rats by contrast with the low-dose and high-dose IBO rats combined (0.67°±.04°C vs 0.51°±.04°C, respectively; p<.05; Fig. 7A), although not by contrast with the two lesion groups separately. Similar daily rhythms and group difference was also evident in DD (0.68°±.06° vs. 0.53°±.04°C, respectively; p<.05; Fig. 6B, 7A).

The *T*_b_ daily waveforms were markedly altered by the feeding schedules. During daytime restricted feeding, all three groups exhibited a relative hypothermia during the first 3–4 h of the light period, with mean *T*_b_ declining into the 36.0–36.4° range, or about 0.4–0.8°C below normal values for that time of day (Fig. 6C,E). During the 3 h prior to the daily meal at ZT6, group mean *T*_b_ increased monotonically in all 3 groups. Within the first 30 min of meal onset, *T*_b_ increased rapidly by another ∼0.7°C, before briefly settling by ∼0.2°C. This was followed by a gradual increase of ∼0.3–0.5°C over the remaining 2.5 h of food access. All rats in all groups exhibited this pattern, including the rat with the largest DMH lesion (Fig. 2L).

During the 2 days of total food deprivation, the *T*_b_ daily waveforms were virtually identical to the waveforms during scheduled feeding, except for the absence of the initial rapid rise of *T*_b_ that occurred during the first 30 min of food access. *T*_b_ declined abruptly at the end of the mealtime, during restricted feeding and during the total food deprivation days. During the nighttime restricted feeding schedule, *T*_b_ again exhibited a monotonic increase prior to mealtime in all intact and DMH ablated rats, and this pattern persisted during 2 days of total food deprivation. Daytime hypothermia was absent during the nocturnal feeding schedule.

Although there were no group differences in the effects of scheduled feeding on the waveforms of the *T*_b_ rhythms, ANOVA revealed a significant effect of both group (F_(2,156)_ = 9.20, p = .0002) and feeding condition (F_(5,156)_ = 14.43, p<.0001) on average daily *T*_b_ (Fig. 7B). During *ad-lib* food access in LD and DD, mean daily *T*_b_ was significantly lower in the two IBO lesion groups relative to the control group (p<.05). Group differences were not significant during restricted feeding (Fig. 6, 7B). Across feeding conditions, mean daily *T*_b_ in each group were significantly lower during the food deprivation tests relative to *ad-lib* food access. There was also a trend for lower mean daily *T*_b_ during daytime scheduled feeding relative to nighttime scheduled feeding (Fig. 7B).

## Discussion

The landmark discovery of a retinorecipient, light-entrainable master circadian pacemaker in the SCN suggested by analogy that SCN-independent food-entrainable oscillators hypothesized to drive food anticipatory circadian rhythms might be similarly located in the hypothalamus, possibly in a single structure containing neurons responsive to nutrients or other feeding-related signals. Lesion studies of medial and lateral hypothalamic structures failed to identify a critical locus [6], [36] until a report that neurotoxic (axon sparing) lesions in the DMH strongly attenuated food-anticipatory behavioral and *T*_b_ rhythms in rats [16]. All of the lesions in that study were partial, and all of the ablated rats showed at least some anticipatory activity, but a significant correlation between the amount of DMH damage and the food anticipation ratios supported an inference that complete lesions would have eliminated anticipatory rhythms altogether. This was the basis for the conclusion that the DMH is ‘critical’ for food anticipatory rhythms. A prediction from this conclusion is that complete removal of the DMH using electrolytic or radiofrequency lesion methods, which kill all cells and fibers of passage, would eliminate anticipatory rhythms. The results of two studies failed to confirm that prediction, and showed instead robust food anticipatory rhythms in rats with very large lesions that unambiguously removed the entire DMH, based on the absence of medial hypothalamic tissue bilaterally between the fornix and the 3^rd^ ventricle, from the top of the 3^rd^ ventricle extending ventrally to the middle of the ventromedial hypothalamus [23], [24]. The presence of clear neuroanatomical landmarks, including the fornix, mammilothalamic tract and ventromedial hypothalamus, eliminated any doubt that the DMH was entirely removed. Differences in cage design, recording method, and feeding procedures were ruled out as relevant [24], [25]. That left the seeming paradox that partial DMH lesions created with a neurotoxin can markedly attenuate food anticipatory rhythms, while total DMH removal by radiofrequency current does not.

The neurotoxin ibotenic acid kills only a subpopulation of neurons and spares fibers of passage, while radiofrequency lesions destroy all neurons, glia and fibers of passage indiscriminately. Taken together, the results indicate that the DMH cannot be the exclusive site of food-entrainable oscillators sufficient to produce robust food anticipatory rhythms, and suggest that fibers of passage through this area may play some role in the expression of these rhythms. This led to a working hypothesis that fibers of passage damaged by very large radiofrequency DMH lesions might include direct or polysynaptic projections from the SCN pacemaker that are responsible for inhibiting activity and arousal during the daily sleep period (lights-on) in nocturnal rats. If DMH neurons played a role inhibiting SCN output, then selective removal of DMH neurons by neurotoxin would attenuate food anticipatory activity, while removal of DMH neurons and SCN outputs concurrently by large radiofrequency lesions would leave food anticipatory rhythms largely spared. According to this model, food anticipatory rhythms in rats with neurotoxic DMH lesions should be restored by removing the SCN or by scheduling food at night, when the SCN does not oppose the expression of activity.

The present study therefore had two objectives. The first objective was to duplicate the recording (radiotelemetry), feeding and lesion methods used by Gooley et al [16] to determine if the results of that study could be replicated. The results do confirm that DMH lesions can attenuate daytime food anticipatory activity, but do not confirm a loss of food anticipatory increases in *T*_b_. The second objective was to test the prediction that attenuation of food anticipatory activity to daytime meals in DMH lesioned rats could be corrected by shifting mealtime to the night. The results are consistent with that prediction, although group mean differences between lesion and intact rats in the magnitude of food anticipatory rhythms in the day and the night were not great.

Using two different volumes of 10% ibotenic acid, we were able to create DMH lesions varying in size from 10–100% complete. These lesions had modest but significant effects on activity and *T*_b_ relative to intact rats. When food was available *ad-libitum*, total daily locomotor activity was lower in high-dose IBO rats, and nocturnality was lower in both IBO lesion groups. Average daily *T*_b_ was also significantly lower in the lesion groups, as was the day-night *T*_b_ difference. These effects on activity and *T*_b_ are consistent with those reported in previous DMH lesion studies using radiofrequency [24] or ibotenic acid lesion methods [35]. Group differences in average daily activity were also evident during the restricted feeding schedules, while *T*_b_ differences between groups were minimal.

Despite the group differences evident in the mean levels of activity and *T*_b_ during *ad-libitum* food access, group differences in food anticipatory activity (anticipation ratios) or in the premeal rise of *T*_b_ during daytime or nightime restricted feeding schedules were not statistically significant. Nonetheless, a subset of 6 rats with DMH lesions exhibited low anticipation ratios relative to baseline during the daytime feeding schedule, and in all cases the magnitude of these ratios improved during nightime feeding schedules to within the range exhibited by intact rats. These results are as predicted if the DMH participates in the expression of food anticipatory activity by exerting a time-of-day (circadian phase) -dependent inhibitory effect on sleep-promoting outputs from the light-entrained SCN pacemaker. The other 8 rats with DMH lesions exhibited anticipation ratios during restricted daytime feeding that were within the range exhibited by intact rats. This presumably reflects sparing of a sufficient population of DMH neurons.

An instructive individual case is that of the rat with the largest lesion, in which the DMH was completely destroyed (Fig. 1C, 2D). This rat exhibited the lowest food anticipation ratio during daytime feeding, and marked improvement of the ratio relative to baseline during the nighttime feeding schedule. While this result supports the hypothesis that the DMH may play a modulatory role in the expression of food anticipation, it does not support a conclusion that the integrity of the DMH is critical either for food anticipatory behavior or a premeal rise of *T*_b_. Food anticipation ratios for this rat, although lower than for other rats, were significantly different from *ad-libitum* baseline days by the second 5-day block of daytime restricted feeding. Furthermore, the anticipation rhythm persisted in DD when meals were omitted for 2 consecutive days. Although the raster style plots for this rat (and even for some intact rats, e.g., Fig. 2B) suggest low amplitude food anticipatory rhythmicity at best, average waveforms and anticipation ratios reveal a persisting ability to coordinate behavior and physiology (*_Tb_*) with predictable daily mealtimes. Whatever role the DMH might play in this function, it is not essential.

Another notable detail in the data from this rat is that during the last 5-day block of daytime food restriction, *T*_b_ rose by ∼1°C from ZT2 (2 h after lights-on) to ZT6 (meal delivery time), as it did in the intact rats. However, within this 4 h time block, *T*_b_ decreased slightly from ZT4-5 (Fig. 2L). If only the 2–3 h immediately before mealtime are used to describe *T*_b_, without presenting full 24 h waveforms from both the food restriction and the *ad-libitum* food access conditions, the results may be misleading. Inspection of full 24 h waveforms for this rat, and for the group data, clearly shows that across hours 2–6 of the light period, *T*_b_ does rise steeply when DMH lesion rats are fed at ZT6, by contrast with the *T*_b_ waveform during ad-lib food access.

The lack of effect of even complete DMH ablation on the early daytime hypothermia and the subsequent premeal rise of *T*_b_ prior to a daily feeding at ZT6 is consistent with the results of other studies showing that the DMH does not participate critically in metabolic and peripheral clock adaptations to restricted feeding schedules. A lower average *T*_b_ during caloric restriction is adaptive by minimizing energy expenditure, and evidently this response is not dependent on the DMH. Another study has shown that resetting of circadian oscillators in peripheral organs is not affected by very large DMH lesions that included significant damage to the ventromedial hypothalamus and arcuate nucleus [37].

The results of this study confirm that the DMH does not contain circadian oscillators critical for driving food anticipatory rhythms of activity or *T*_b_, but might facilitate expression of food entrainable oscillators located elsewhere in the brain by actively inhibiting outputs from the SCN pacemaker that normally promote sleep and inhibit activity in the day. These conclusions are consonant with the results and conclusions of another study that demonstrated a neural basis for inhibition of the SCN by the DMH, and that provided evidence for recovery of attenuated food anticipatory rhythms in DMH lesioned rats by subsequent removal of the SCN [30]. Although some DMH neurons express daily rhythms of circadian clock gene expression in nocturnal rats and mice on daytime restricted feeding schedules, clock genes in this area can be induced by food deprivation alone, and by randomized feeding schedules that do not generate food anticipatory rhythms [17], [38], [39]. These observations are consistent with a role for DMH neurons as sensors of caloric restriction that modulate SCN output during the day, to enable hungry animals to exploit the daytime temporal niche when nutritional needs are not being met by foraging at night. These results underscore the potential importance of employing both daytime and nightime restricted feeding schedules to thoroughly phenotype food anticipatory rhythms in rats with neural or genetic deficiencies.
